# Dichloridobis(*N*,*N*,*N*′,*N*′-tetra­methyl­thio­urea-κ*S*)mercury(II)

**DOI:** 10.1107/S1600536810028138

**Published:** 2010-07-17

**Authors:** Sidra Nawaz, Haseeba Sadaf, Mohammed Fettouhi, Atif Fazal, Saeed Ahmad

**Affiliations:** a Department of Chemistry, University of Engineering and Technology, Lahore 54890, Pakistan; b Department of Chemistry, King Fahd University of Petroleum and Minerals, Dhahran 31261, Saudi Arabia

## Abstract

In the title compound, [HgCl_2_(C_5_H_12_N_2_S)_2_], the Hg^II^ atom is located on a twofold rotation axis and is bonded in a distorted tetra­hedral coordination mode to two chloride ions and to two tetra­methyl­thio­urea (tmtu) mol­ecules through their S atoms. The crystal structure is stabilized by C—H⋯N and C—H⋯S hydrogen bonds.

## Related literature

For background to Hg(II) complexes with thio­urea ligands, see: Ahmad *et al.* (2009 ▶); Chieh (1977 ▶); Lobana *et al.* (2008 ▶); Popovic *et al.* (2000 ▶, 2002 ▶). The structure of the title compound is isotypic with [Cd(tmtu)_2_Br_2_] (Nawaz *et al.*, 2010*a* ▶) and [Cd(tmtu)_2_I_2_] (Nawaz *et al.*, 2010*b* ▶).
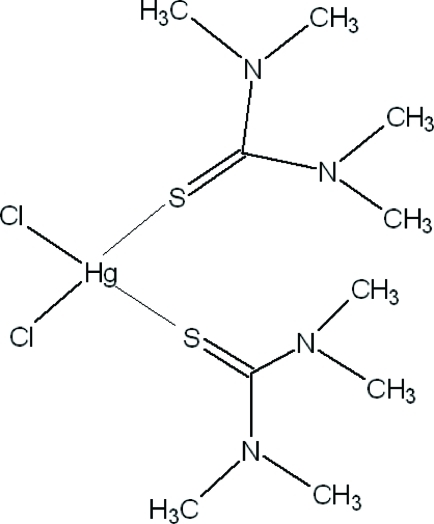


## Experimental

### 

#### Crystal data

[HgCl_2_(C_5_H_12_N_2_S)_2_]
*M* *_r_* = 535.94
Monoclinic, 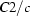

*a* = 18.7418 (12) Å
*b* = 9.5920 (6) Å
*c* = 13.5177 (9) Å
β = 130.834 (1)°
*V* = 1838.6 (2) Å^3^
*Z* = 4
Mo *K*α radiation
μ = 8.88 mm^−1^
*T* = 293 K
0.29 × 0.24 × 0.11 mm

#### Data collection

Bruker SMART APEX area detector diffractometer
Absorption correction: multi-scan (*SADABS*; Sheldrick, 1996 ▶) *T*_min_ = 0.183, *T*_max_ = 0.442
12167 measured reflections
2281 independent reflections
2103 reflections with *I* > 2σ(*I*)
*R*_int_ = 0.031

#### Refinement

*R*[*F* ^2^ > 2σ(*F* ^2^)] = 0.020
*wR*(*F* ^2^) = 0.040
*S* = 1.07
2281 reflections
92 parameters
H-atom parameters constrained
Δρ_max_ = 0.72 e Å^−3^
Δρ_min_ = −0.79 e Å^−3^

### 

Data collection: *SMART* (Bruker, 2008 ▶); cell refinement: *SAINT* (Bruker, 2008 ▶); data reduction: *SAINT*; program(s) used to solve structure: *SHELXS97* (Sheldrick, 2008 ▶); program(s) used to refine structure: *SHELXL97* (Sheldrick, 2008 ▶); molecular graphics: *SHELXTL* (Sheldrick, 2008 ▶); software used to prepare material for publication: *SHELXTL*.

## Supplementary Material

Crystal structure: contains datablocks I, global. DOI: 10.1107/S1600536810028138/wm2376sup1.cif

Structure factors: contains datablocks I. DOI: 10.1107/S1600536810028138/wm2376Isup2.hkl

Additional supplementary materials:  crystallographic information; 3D view; checkCIF report

## Figures and Tables

**Table 1:** Selected bond lengths (Å)

Hg1—Cl1	2.5028 (8)
Hg1—S1	2.5329 (7)

**Table 2:** Hydrogen-bond geometry (Å, °)

*D*—H⋯*A*	*D*—H	H⋯*A*	*D*⋯*A*	*D*—H⋯*A*
C2—H2*A*⋯N2	0.96	2.52	2.849 (6)	100
C3—H3*A*⋯S1	0.96	2.68	2.996 (6)	100
C5—H5*A*⋯S1	0.96	2.62	3.024 (5)	105

**Fig. 1.**
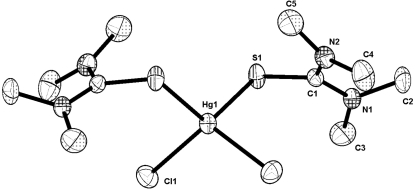
The molecular structure of title compound with atomic numbering scheme. Displacement ellipsoids are drawn at the 30% probability level. H-atoms were omitted for clarity.

## supplementary crystallographic information

### Comment

The coordination chemistry of mercury(II) complexes with thiourea type ligands has been the subject of several recent studies because of the importance of such systems as structural models in biology (Popovic *et al.*, 2000; 2002). Mercury(II) is known form a wide variety of 1:1 and 1:2 complexes of the types *L*Hg*X*_2_ (Popovic *et al.*, 2002) and *L*_2_Hg*X*_2_ (Ahmad *et al.*, 2009; Chieh, 1977: Lobana *et al.*, 2008), where *X* is a halide or pseudohalide, having structural arrangements entirely based on tetrahedral or pseudo-tetrahedral environments. We have recently reported the crystal structure of a Hg(CN)_2_ complex of *N*,*N*'-dibutylthiourea (dbtu) (Ahmad *et al.*, 2009). Herein we report on the crystal structure of a mercury(II) chloride complex of tetramethylthiourea (tmtu), [Hg(C_5_H_12_N_2_S_2_)_2_Cl_2_], (I) .

The crystal structure of (I) consists of discrete molecular species in which the mercury atom is located on a twofold rotation axis (Fig. 1) and is bonded in a distorted tetrahedral coordination mode to two chloride ions and to two tetramethylthiourea (tmtu) molecules. The Hg—S and Hg—Cl bond lengths are 2.5329 (7) and 2.5028 (8) Å, respectively. The bond angles around Hg are in the range expected for a tetrahedral coordination, with the S—Hg—S angle (120.75 (4)°) having the largest deviation from the ideal value. The main cause of this deviation is the steric interaction between the —CH_3_ groups. The SCN_2_— moiety of Tmtu is essentially planar with the C—N and C—S bond lengths corresponding to the values intermediate between single and double bonds.

The structure of the title compound is isotypic with [Cd(tmtu)_2_Br_2_] (Nawaz *et al.*, 2010*a*) and [Cd(tmtu)_2_I_2_] (Nawaz *et al.*, 2010*b*).

For a more detailed description of the structure, see: Nawaz *et al.* (2010*a*).

### Experimental

To 0.27 g (1.0 mmol) mercury(II) chloride in 10 ml methanol was added two equivalents of tetramethylthiourea in 15 ml methanol. A clear solution was obtained that was stirred for 30 minutes. The colorless solution was filtered and the filtrate was kept at room temperature for crystallization. As a result, a white crystalline product was obtained, that was finally washed with methanol and dried.

### Refinement

H atoms were placed in calculated positions with a C—H distance of 0.96 Å and *U*_iso_(H) = 1.5 *U*_eq_(C).

### Crystal data

**Table:**

[HgCl_2_(C_5_H_12_N_2_S)_2_]	*F*(000) = 1032
*M**_r_* = 535.94	*D*_x_ = 1.936 Mg m^−^^3^
Monoclinic, *C*2/*c*	Mo *K*α radiation, λ = 0.71073 Å
Hall symbol: -C 2yc	Cell parameters from 12167 reflections
*a* = 18.7418 (12) Å	θ = 2.6–28.3°
*b* = 9.5920 (6) Å	µ = 8.88 mm^−^^1^
*c* = 13.5177 (9) Å	*T* = 293 K
β = 130.834 (1)°	Colourless, plate
*V* = 1838.6 (2) Å^3^	0.29 × 0.24 × 0.11 mm
*Z* = 4	

### Data collection

**Table:**

Bruker SMART APEX area detector diffractometer	2281 independent reflections
Radiation source: normal-focus sealed tube	2103 reflections with *I* > 2σ(*I*)
graphite	*R*_int_ = 0.031
ω scans	θ_max_ = 28.3°, θ_min_ = 2.6°
Absorption correction: multi-scan (*SADABS*; Sheldrick, 1996)	*h* = −24→24
*T*_min_ = 0.183, *T*_max_ = 0.442	*k* = −12→12
12167 measured reflections	*l* = −18→18

### Refinement

**Table:**

Refinement on *F*^2^	Secondary atom site location: difference Fourier map
Least-squares matrix: full	Hydrogen site location: inferred from neighbouring sites
*R*[*F*^2^ > 2σ(*F*^2^)] = 0.020	H-atom parameters constrained
*wR*(*F*^2^) = 0.040	*w* = 1/[σ^2^(*F*_o_^2^) + (0.0109*P*)^2^ + 2.5249*P*] where *P* = (*F*_o_^2^ + 2*F*_c_^2^)/3
*S* = 1.07	(Δ/σ)_max_ = 0.002
2281 reflections	Δρ_max_ = 0.72 e Å^−^^3^
92 parameters	Δρ_min_ = −0.79 e Å^−^^3^
0 restraints	Extinction correction: *SHELXL97* (Sheldrick, 2008), Fc^*^=kFc[1+0.001xFc^2^λ^3^/sin(2θ)]^-1/4^
Primary atom site location: structure-invariant direct methods	Extinction coefficient: 0.00244 (8)

### Special details

**Table:**

Geometry. All e.s.d.'s (except the e.s.d. in the dihedral angle between two l.s. planes) are estimated using the full covariance matrix. The cell e.s.d.'s are taken into account individually in the estimation of e.s.d.'s in distances, angles and torsion angles; correlations between e.s.d.'s in cell parameters are only used when they are defined by crystal symmetry. An approximate (isotropic) treatment of cell e.s.d.'s is used for estimating e.s.d.'s involving l.s. planes.
Refinement. Refinement of *F*^2^ against ALL reflections. The weighted *R*-factor *wR* and goodness of fit *S* are based on *F*^2^, conventional *R*-factors *R* are based on *F*, with *F* set to zero for negative *F*^2^. The threshold expression of *F*^2^ > σ(*F*^2^) is used only for calculating *R*-factors(gt) *etc*. and is not relevant to the choice of reflections for refinement. *R*-factors based on *F*^2^ are statistically about twice as large as those based on *F*, and *R*- factors based on ALL data will be even larger.

### Fractional atomic coordinates and isotropic or equivalent isotropic displacement parameters (Å^2^)

**Table:**

	*x*	*y*	*z*	*U*_iso_*/*U*_eq_	
Hg1	1.0000	0.703186 (17)	0.2500	0.04539 (7)	
Cl1	1.14323 (5)	0.55933 (9)	0.34397 (8)	0.0591 (2)	
S1	1.02994 (5)	0.83371 (9)	0.43697 (7)	0.04983 (18)	
N1	0.91286 (18)	0.7622 (3)	0.4747 (3)	0.0482 (6)	
N2	0.84430 (16)	0.8798 (3)	0.2830 (2)	0.0488 (6)	
C1	0.92029 (18)	0.8240 (3)	0.3933 (3)	0.0367 (5)	
C2	0.8466 (3)	0.8103 (4)	0.4900 (4)	0.0704 (10)	
H2A	0.8192	0.8971	0.4446	0.106*	
H2B	0.8793	0.8232	0.5812	0.106*	
H2C	0.7977	0.7421	0.4542	0.106*	
C3	0.9842 (3)	0.6647 (4)	0.5756 (4)	0.0769 (11)	
H3A	1.0131	0.6182	0.5468	0.115*	
H3B	0.9549	0.5970	0.5914	0.115*	
H3C	1.0313	0.7149	0.6548	0.115*	
C4	0.7491 (2)	0.8240 (5)	0.2114 (4)	0.0802 (12)	
H4A	0.7531	0.7329	0.2441	0.120*	
H4B	0.7174	0.8179	0.1200	0.120*	
H4C	0.7144	0.8847	0.2231	0.120*	
C5	0.8518 (3)	0.9798 (4)	0.2094 (4)	0.0774 (11)	
H5A	0.9121	1.0250	0.2669	0.116*	
H5B	0.8027	1.0483	0.1711	0.116*	
H5C	0.8454	0.9323	0.1414	0.116*	

### Atomic displacement parameters (Å^2^)

**Table:**

	*U*^11^	*U*^22^	*U*^33^	*U*^12^	*U*^13^	*U*^23^
Hg1	0.05014 (10)	0.04849 (10)	0.05259 (11)	0.000	0.04018 (9)	0.000
Cl1	0.0519 (4)	0.0599 (5)	0.0652 (5)	0.0126 (3)	0.0381 (4)	0.0065 (4)
S1	0.0386 (3)	0.0716 (5)	0.0453 (4)	−0.0091 (3)	0.0300 (3)	−0.0142 (3)
N1	0.0611 (15)	0.0488 (13)	0.0566 (14)	0.0005 (11)	0.0480 (13)	0.0002 (11)
N2	0.0439 (13)	0.0560 (15)	0.0465 (13)	0.0051 (11)	0.0295 (11)	0.0020 (11)
C1	0.0415 (13)	0.0364 (13)	0.0415 (13)	−0.0023 (10)	0.0312 (12)	−0.0061 (10)
C2	0.085 (2)	0.081 (3)	0.092 (3)	−0.011 (2)	0.078 (2)	−0.014 (2)
C3	0.098 (3)	0.070 (2)	0.076 (2)	0.015 (2)	0.063 (2)	0.024 (2)
C4	0.0387 (17)	0.116 (3)	0.070 (2)	−0.0013 (18)	0.0292 (17)	−0.015 (2)
C5	0.088 (3)	0.081 (3)	0.069 (2)	0.027 (2)	0.054 (2)	0.029 (2)

### Geometric parameters (Å, °)

**Table:**

Hg1—Cl1^i^	2.5028 (8)	C2—H2B	0.9600
Hg1—Cl1	2.5028 (8)	C2—H2C	0.9600
Hg1—S1	2.5329 (7)	C3—H3A	0.9600
Hg1—S1^i^	2.5329 (7)	C3—H3B	0.9600
S1—C1	1.730 (3)	C3—H3C	0.9600
N1—C1	1.336 (3)	C4—H4A	0.9600
N1—C2	1.460 (4)	C4—H4B	0.9600
N1—C3	1.461 (4)	C4—H4C	0.9600
N2—C1	1.327 (3)	C5—H5A	0.9600
N2—C5	1.453 (4)	C5—H5B	0.9600
N2—C4	1.466 (4)	C5—H5C	0.9600
C2—H2A	0.9600		
			
Cl1^i^—Hg1—Cl1	113.08 (4)	H2A—C2—H2C	109.5
Cl1^i^—Hg1—S1	104.08 (3)	H2B—C2—H2C	109.5
Cl1—Hg1—S1	107.56 (3)	N1—C3—H3A	109.5
Cl1^i^—Hg1—S1^i^	107.56 (3)	N1—C3—H3B	109.5
Cl1—Hg1—S1^i^	104.08 (3)	H3A—C3—H3B	109.5
S1—Hg1—S1^i^	120.75 (4)	N1—C3—H3C	109.5
C1—S1—Hg1	101.20 (9)	H3A—C3—H3C	109.5
C1—N1—C2	122.2 (3)	H3B—C3—H3C	109.5
C1—N1—C3	121.9 (3)	N2—C4—H4A	109.5
C2—N1—C3	114.4 (3)	N2—C4—H4B	109.5
C1—N2—C5	121.5 (3)	H4A—C4—H4B	109.5
C1—N2—C4	122.9 (3)	N2—C4—H4C	109.5
C5—N2—C4	114.2 (3)	H4A—C4—H4C	109.5
N2—C1—N1	119.5 (2)	H4B—C4—H4C	109.5
N2—C1—S1	121.6 (2)	N2—C5—H5A	109.5
N1—C1—S1	118.9 (2)	N2—C5—H5B	109.5
N1—C2—H2A	109.5	H5A—C5—H5B	109.5
N1—C2—H2B	109.5	N2—C5—H5C	109.5
H2A—C2—H2B	109.5	H5A—C5—H5C	109.5
N1—C2—H2C	109.5	H5B—C5—H5C	109.5

Symmetry codes: (i) −*x*+2, *y*, −*z*+1/2.

### Hydrogen-bond geometry (Å, °)

**Table:**

*D*—H···*A*	*D*—H	H···*A*	*D*···*A*	*D*—H···*A*
C2—H2A···N2	0.96	2.52	2.849 (6)	100
C3—H3A···S1	0.96	2.68	2.996 (6)	100
C5—H5A···S1	0.96	2.62	3.024 (5)	105
